# Molecular and epidemiological characterization of *Plasmodium vivax* recurrent infections in southern Mexico

**DOI:** 10.1186/1756-3305-6-109

**Published:** 2013-04-18

**Authors:** Lilia Gonzalez-Ceron, Jianbing Mu, Frida Santillán, Deirdre Joy, Marco A Sandoval, Gerardo Camas, Xinzhuan Su, Elena V Choy, Rene Torreblanca

**Affiliations:** 1 CRISP, National Institute for Public Health, Tapachula, Chiapas, 30700, Mexico; 2 Laboratory of Malaria and Vector Research, NIAID, National Institutes of Health, Bethesda, MD, 20892, USA; 3 Sanitary Jurisdiction VII, Ministry of Health, Tapachula, Chiapas, Mexico

**Keywords:** *Plasmodium vivax*, Hypnozoite, Relapse, Genetic homology, Epidemiology, Southern Mexico

## Abstract

**Background:**

In southern Mexico, malaria transmission is low, seasonal, and persistent. Because many patients are affected by two or more malaria episodes caused by *Plasmodium vivax*, we carried out a study to determine the timing, frequency, and genetic identity of recurrent malaria episodes in the region between 1998 and 2008.

**Methods:**

Symptomatic patients with more than one *P. vivax* infection were followed up, and blood samples were collected from primary and recurrent infections. DNA extracted from infected blood samples was analyzed for restriction fragment length polymorphism (RFLP) in genes encoding *csp* and *msp3α*, as well as size variation in seven microsatellites.

**Results:**

One hundred and forty six parasite samples were collected from 70 patients; of these, 65 patients had one recurrent infection, four had two, and one had three recurrent infections. The majority of recurrent infections occurred within one year of the primary infection, some of which were genetically homologous to the primary infection. As the genetic diversity in the background population was high, the probability of homologous re-infection was low and the homologous recurrences likely reflected relapses. These homologous recurrent infections generally had short (< 6 months) or long (6–12 months) intervals between the primary (PI) and recurrent (RI) infections; whereas infections containing heterologous genotypes had relatively longer intervals. The epidemiological data indicate that heterologous recurrences could be either relapse or re-infections.

**Conclusions:**

Genetic and temporal analysis of *P. vivax* recurrence patterns in southern Mexico indicated that relapses play an important role in initiating malaria transmission each season. The manifestation of these infections during the active transmission season allowed the propagation of diverse hypnozoite genotypes. Both short- and long-interval relapses have contributed to parasite persistence and must be considered as targets of treatment for malaria elimination programs in the region to be successful.

## Background

*Plasmodium vivax* is responsible for 99% of malaria cases in Mexico*.* In the 1980’s more than 100,000 cases were reported each year. Due to intensive malaria control efforts carried out by the National Malaria Control Program in the 1990s, the numbers of cases have been declining ever since. Malaria transmission is general low and seasonal, and is concentrated in residual foci along the Pacific coast. In the past 10 years, ~80% of malaria cases reported in Mexico have been from Oaxaca and Chiapas provinces [1,2]. These malaria foci are vulnerable to severe climate events as evidenced by malaria outbreaks that occurred coincident with “el Niño” and hurricane Paulina in 1998 in Oaxaca and Chiapas States, and Hurricane Stan at the end of 2005 in southern Chiapas.

*P. vivax* relapses are recurrent blood infections produced by latent parasites (hypnozoites) in the liver [3]. These episodes are of epidemiological significance, as they contribute to the persistence of malaria transmission in affected regions, which may be particularly relevant where malaria transmission is seasonal. A person inoculated with *P. vivax* sporozoites has from 5 to 80% probability of producing latent hypnozoites after the primary attack [4]. These latent parasites are activated at variable latency periods by as yet unknown factors [5,6]. The relapse pattern comprises the latency period and number of relapse episodes; it may be influenced by parasite strain, transmission dynamics, sporozoite inoculation rate for different vector species, and host factors [5-8].

Primaquine (PQ) is an 8-aminoquinoline and is the only licensed antimalarial drug currently available that can be used to treat the dormant liver stage parasites (hypnozoites). PQ has been used to prevent *P. vivax* relapses in 1950 [9]. A 14-day PQ regimen (0.25 mg base/kg/day) is currently recommended by the World Health Organization (WHO) [10]. In some *P. vivax* affected regions, parasites show reduced susceptibility to supervised PQ treatment, and higher PQ doses are required to reduce recurrence rates [11-13]. In Mexico, standard anti-malarial treatment guidelines included intermittent single doses of chloroquine (CQ) and PQ combination adjusted to age groups (from 10 and 0.75 for adults to 2.5 and ~0.16 mg/kg for children, respectively) [14] as recommended by WHO [10], is administered once monthly for three months, then no treatment is given for the following three months. This treatment regimen is repeated up to six times for a total of three years to suppress recurrence onset [14,15]. Although this regimen has been administered for more than a decade, there is no baseline data describing the recurrence characteristics prevailing in southern Mexico. Currently, there is no evidence suggesting the presence of chloroquine resistant *P. vivax* in the region.

In recent years, Mexico has been in a pre-elimination phase due to significant reductions in malaria transmission [16]. It is now imperative to determine prevailing relapse patterns in the affected regions in order to support effective pre-elimination practices. Relapse patterns could evolve in response to changing drug pressure and transmission dynamics. The genetic identity of relapsing parasites is known to vary according to the transmission intensity [17-19]. In very low transmission settings most individuals are inoculated by no more than one infective mosquito bite per year, often carrying a single genotype infection, and the resulting primary and relapse episodes are mostly caused by the same parasite genotype (genetic homology) [17]. However, under more intense and complex transmission where mixed genotype infections and multiple infected bites are more common, many relapse episodes may in fact be produced by parasites with genotypes not initially detected in the primary infection (genetic heterology) [18,19]. Additionally, it has been proposed that relapse could be triggered by re-infection, leading to frequent infections of mixed genotypes [5]. In fact, one would predict a spectrum of homologous versus heterologous relapse rates across different transmission settings. In this report, molecular and epidemiological analyses of recurrent malaria infections were carried out in a malaria hypo-endemic region of southern Chiapas between 1998 and 2008 in order to understand the timing and molecular identity of recurrent *P. vivax*.

## Methods

### Sample collection

The study site (Tapachula and surrounding municipalities in southern Mexico) comprises two main sub-regions with alternating malaria transmission; in the temperate foothills transmission occurs mainly during January-June and in the coastal region during August-November. Symptomatic patients who consented to donate a capillary or venous blood sample were enrolled in the study. *P. vivax* infection was diagnosed by Giemsa-stained thick blood smears at the Regional Center for Research in Public Health (CRISP-INSP) during 1998–2008 [20-22]. Whole blood was stored in liquid nitrogen, and then transferred to Whatman No.2 filter paper (Whatman International, Ltd., Maidstone, England), dried and kept in the dark. After collection, filter paper samples were coded to block out identifying patient information, and part of the paper was used in DNA extraction and microsatellite analyses. All patients were treated by the local malaria control program according to the Mexican Malaria Treatment Guidelines [14]. Patients received anti-malarial treatment whether or not they agreed to give a blood sample. For the relapse study, both passive and active follow up were carried out. A second blood sample was collected from symptomatic patients who returned to CRISP-INSP seeking diagnosis and treatment of recurrent *P. vivax* infection (1998–2008). In addition, other patients were visited at their villages monthly to detect recurrent symptomatic infections (2005–2008). The majority of the analyses reported here were carried out on the paired blood samples with primary (PI) and recurrent (RI) infections from a single patient. However, we also included available non-paired samples from the region in the estimate of background microsatellite genetic diversity. The interval of infection-time between primary and recurrent infections is reported in weeks.

### Molecular analysis of *P. vivax* samples

One hundred μl of infected blood or 6 punches of 5 mm diameter dried blood in filter paper were used to extract DNA using the QIAamp® DNA Blood Mini Kit (QIAGEN, Hilden, Germany) following the manufacturer’s instructions. The DNA obtained was dissolved in water and stored at −20°C until used. To determine the *P. vivax* genotypes, nine distinct markers (three marker sets) were analyzed, including variants in the circumsporozoite repeat region (*cspr*) [23] and merozoite surface protein 3 alpha domain (*msp3α*) [24-26], and 7 microsatellites positioned on different chromosomes (Table 1) [27]. The *cspr* and *msp3α* variants were genotyped by polymerase chain reaction and restriction fragment length polymorphism (PCR-RFLP) [23]. The microsatellite markers were genotyped using capillary electrophoresis.

**Table 1:** ***P. vivax *microsatellites and their primers used for PCR amplification and detection of nucleotide fragment length**

**Microsatellite**	**Chromosome**	**Repeat unit**	**Primers**	
*MS* 040	6	AAAT	F	*6FAM-*ATTTGCGTACGGTTAAGAT
			R	CAGGGTTATTCAATTTGCT
*MS* 092	5	GAA	F	*6FAM-*TCACTGATCTTTTCGCATG
			R	TAGTAGCATAGTGGTAGTA
*MS* 116	10	GAA	F	*NED-*AAATGCAAGATCCAAGAAAT
			R	GTCGCTCTTCATGTGGCA
*MS* 033	3	CAT	F	*6FAM-*CGATTCGTGCTATTTGC
			R	CCTGCTACATATTTGGC
*MS* 176	14	AT	F	*6FAM-*ATAATGGCGTCATCCTTCA
			R	TTCAGCATGCGCTGTTTAT
*MS* 315	3	AT	F	*NED-*TTAACGGTTAATCCTCTATT
			R	TTGTGTCTATTTGGCCATT
*MS* 206	12	GTT	F	*6FAM-*TCTTTATGTTGTACTGCTC
			R	ACCACTTACAAAAGTGTGA

MS, microsatellite; F, forward; R, reverse.

*a)* PCR-RFLP genotyping of the *csp* central repeat region (*cspr*)*.* A nested PCR was carried out to amplify the complete *cspr* using primers CSP-1 (5’-cgcactgcgggcacaatgtagatc-3’) and CSP-2 (5’-ggttacactgcatggagtcc-3’) for the first round amplification, and F-Pv9a (5´-gccaacggtagctctaacttt-3´) [28] and CSP-RI (5'-aataagctgaaacaacca-3') for the second amplification. The PCR reaction mixed was prepared as follow: 2 μl 10X Buffer, 2 mM magnesium chloride, 0.2 mM dNTPs (Invitrogen, 10297018 Carlsbad, CA.), 10 μM each primer, 5 U GoTaq® Flexi DNA polymerase (Promega, Madison WI) and approximately 100 ng of genomic DNA in a 20 μl final volume. Two μl of the product from the first amplification was added to the second PCR of the same setup. The PCR conditions were as follow: first cycle at 95°C for 3 min, followed by 35 cycles of 95°C for 30 sec, 57°C (for first PCR) or 58°C (for second PCR) for 30 sec and 72°C for 1 min, and followed by a final extension at 72°C for 10 min using a MyCycler (Biorad, Hercules). The PCR products were digested with *Alu*I (New England Biolabs, Beverly, MA) and *BstI* (Promega, Madison WI) which have restriction sites in the *cspr* encoding Vk247 and Vk210 regions, respectively [29]. DNA fragments were resolved in a 1.5% gel, visualized under a UV-transilluminator and photographed using the BioDoc-it™ digital photo-documentation system (UVP Inc, Upland**,** California).

*b)* PCR-RFLP genotyping of the *msp3α* gene*.* The *msp3α* gene was amplified in a nested PCR using the published primers (P1, P2, N1 and N2) and a modified version of a previously reported protocol [30]. The PCR was prepared as follow: 2 μl 10X buffer, 1.875 mM magnesium chloride, 0.5 mM of dNTPs (Invitrogen, Carlsbad, CA.), 25 pM of each primer P1 and P2, 1.25 U of GoTaq® Flexi DNA polymerase (Promega, Madison WI) and 1 or 5 μl of genomic DNA extracted from whole or dried blood, respectively, in a final PCR volume of 20 μl. The first PCR was run at 95°C for 3 min, followed by 35 cycles of 94°C for 60 sec, 56°C for 60 sec and 72 °C for 2.5 min, and a final extension cycle at 72°C for 10 min. The second round of PCR reaction was carried out with N1 and N2 primers using the same PCR reaction after addition of 2 μl of first PCR product into a 20 μl mixture. The reaction was run at 95°C for 2 min, followed by 30 cycles of 94°C for 60 sec, 62°C for 60 sec and 72°C for 1.5 min, and a final extension cycle at 72°C for 10 min. For twenty-seven samples that failed to amplify, magnesium chloride and dNTPs were increased to 2.5 mM and 1.0 mM, respectively, and PCR conditions were altered. A total of eight samples did not amplify under either PCR conditions. PCR products were digested with *Alu*I (New England BioLabs, Beverly, MA), which was used to distinguish multiple *msp3α* alleles for parasites from distinct geographic regions such as Papua New Guinea [30], India [31], Thailand [26], and Iran [23]. The digested paired DNA samples were separated in 2% agarose gels and stained with ethidium bromide and photographed.

*c)* Microsatellite (MS). Seven MS were also used to type recurrent infections to validate the capacity of the combined genotype *cspr-msp3α* to discriminate heterologous infections, because MS are generally more variable than nucleotide substitutions. In addition, 236 non-recurrent samples collected during the same time period were also typed using the same MS in order to provide background diversity and marker performance estimates. Samples were scored as mixed genotypes if a MS marker produced a minor peak ≥ 33% of the primary peak. MS primers are listed in Table 1. Due to limited DNA quantity or quality for some samples, we were not able to obtain microsatellite information for all the samples.

### Data analysis

Malaria cases reported by the Sanitary Jurisdiction VII during 1998–2007 were compiled in order to calculate the annual parasitic index, defined as number of cases/1,000 individuals (from affected villages)/per year. From this we estimated the number of cases/1,000 individuals/2-month intervals in order to compare rates between high and low transmission periods. The case report includes all cases confirmed by microscopic analysis of thick smears from all institutions of the corresponding jurisdiction. To characterize the recurrent patterns in the study region, a database was compiled to identify the number of malaria episodes per person indicating name, gender, age, village, municipality of origin and previous malaria infections (self-reported or by a family member). The number of malaria cases diagnosed per two-month period was plotted to simulate transmission dynamics and to identify intervals with minimum risk of re-infection that could be used to identify putative heterologous relapse episodes. For patients identified as having more than one infection, the time-interval between primary and recurrent infections was estimated starting with the day the sample was collected for *P. vivax* diagnosis. Once malaria episodes were associated with individual patients, all samples were coded and all the patient information was masked. The original database was secured at the study investigator’s office. Patients registered between 2005 and 2008 as having more than one episode were confirmed by a home-visit.

For putative relapse identification, blood samples from patients with more than one episode and with good genotype data, comprising at least *cspr* and *msp3α* genotypes, and/or 6–7 MS, and the malaria epidemiology data were used. Recurrent infections showing similar *cspr* and *msp3α* genotypes as the primary infections were defined as homologous. The correspondence between *cspr-msp3α* and MS heterology vs homology was determined. Heterologous recurrent infections detected when transmission was low or non-existent (based on malaria case records) may in fact be heterologous relapse. Pairs of samples with mixed genotype infections detected in either the primary or recurrent infection were not included in the analysis. Relapse episodes were grouped according to their time as follows: 1) early (1–26 weeks), long (27–52 weeks) [32], or late (> 52 weeks). We calculated the ratio of the number of homologous versus heterologous recurrences within 52 weeks and > 52 weeks. All microsatellite analyses were carried out as described [27] using GenAlEx v 6.5 [33].

All MS analyses were blinded to the relationship between samples and their origins. The baseline population diversity was derived from MS genotype data from 326 non-recurrent infections collected during the 10 year study period. The seven MS loci were used in combination to calculate the likelihood that two infections have the same seven-locus genotype by chance by taking the product of the individual homozygosities [34]. Chi-square and t-test (at 95% confidence) were carried out in STATA 9.0 (Stata Corporation LD).

### Ethical approval

The protocol for sample collection was approved by the Ethics Committee of the National Institute of Public Health (INSP), Mexico. Written informed consent was obtained from all patient and/or their guardians for patients below 18 years old.

## Results

### Transmission dynamics and temporal pattern of recurrent malaria cases compiled from the study region during 1998–2007

**Figure 1:**
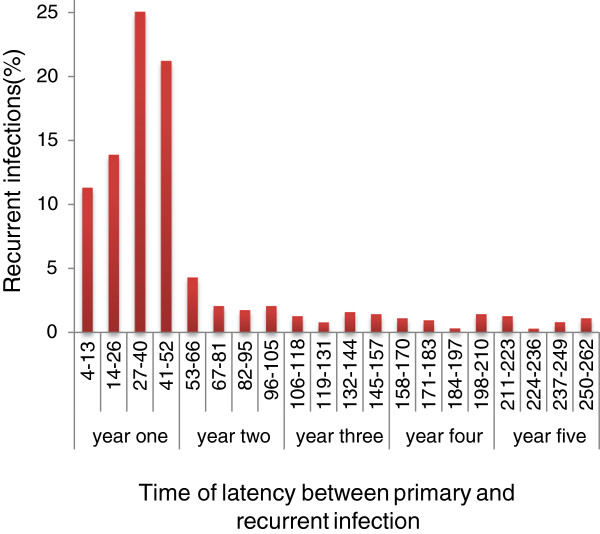
**Distribution of recurrent intervals for *P. vivax *infections.** All recurrent *P. vivax* infections documented in the records of the Malaria Control Program (Jurisdiction VII of Chiapas) during 1998–2007 (n=590, comprising 94% of all the samples excluding those with extreme longer intervals. PI, primary infection; RI, recurrent infection).

During this period, the annual parasitic index (API) fluctuated between 0.58 and 3.4, and between 0.25 and 2 for 1,000 individuals living in the affected villages or the entire municipality, respectively. The minimal number of malaria cases reported per 1,000 individuals/2 months ranged from 0.031 to 0.41 across all years. In contrast, the highest transmission levels occurred during the dry season, with between 0.43 and 1.9 cases per 1,000 individuals/2 months. We therefore set a high transmission lower bound cut-off at ≥ 0.4 cases/1,000 individuals/2 month across the 10 year-period. From 7,694 malaria reports included in the database, 8.1% (n=626) of patients were identified as having one or more recurrent *P. vivax* infections. None of the recurrences occurred within 28 days (CQ surveillance period); all recurrences occurred from 5 weeks to several years after the primary infection. Among those, 25.2% occurred ≤ 26 weeks, 46.3% occurred within 27 and 52 weeks, and 28.4% occurred ≥ 105 weeks after the primary infection (Figure 1). From the total recurrent infections, 88.2% reported two malaria episodes, 9.7% had three malaria episodes, and only a few patients had three to six recurrent episodes.

### *cspr-msp3α* genotypes and genetic diversity

**Figure 2:**
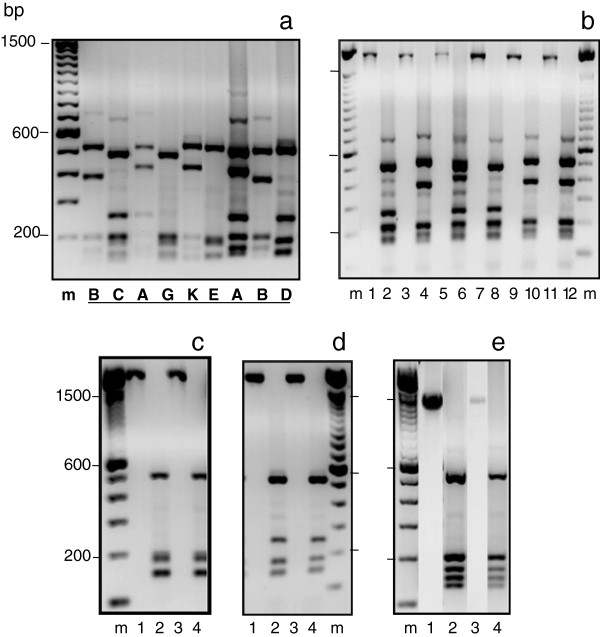
***Plasmodium vivax msp3α *genotypes. **PCR products were digested with *Alu* I, and the digested products are separated side-by-side with their undigested products on agarose gels. **a)** Shows different genotypes indicated by capital letters underneath. **b)** Lanes 1, 3, 5, 7, 9, and 11 are undigested products of ~2.0 kb, and lanes 2, 4, 6, 8, 10, and 12 were the same products digested with *Alu* I. Lane 2 presents genotype C; lane 4, 10, and 12 present genotype B; lane 6 and 8 are mixed genotypes of A+C; **c)** genotype G; **d)** genotype D; and **e)** genotype H. m, 100 bp DNA ladder.

Genotypes from 146 blood samples collected from 70 patients with more than one infection were scored based on the three marker sets to determine whether recurrent infections contained the same genotype(s) as the primary infection (PI), as well as to detect mixed genotype infections (Additional file 1). Among these patients, 65 had one recurrent infection (RI), four had two RI, and one had 3 RI. The *cspr* genotype was determined for all but two samples (one pair with insufficient sample) (Table 2). RFLP-*msp3α* genotypes were successfully obtained for 117 paired samples from a subset of 56 patients. A conserved ~2 kb *msp*3α product was amplified in all these samples except from patient 58 that had a ~1.5 kb fragment. Two *cspr* genotypes (corresponding to vk210 and vk247) and eight *msp3α* genotypes were identified (Figure 2), and a total of 113 single *P. vivax msp*3α genotype infections were detected. Among the eight *msp*3α genotypes, genotype A, B, and C comprised the majority (85.8%). All samples with the *msp*3α-A genotype also had the *cspr* vk247 genotype. Samples with *msp*3α-B or *msp*3α-C had either *cspr* vk210 or vk247 genotypes. There were five other less common *msp*3α genotypes; all have *cspr* vk210. Genotype D was detected in both PI and RI of two patients; E was detected in PI and RI of three patients; whereas G and J were detected in PI and RI from a single patient each (patient 58 and 59 respectively). Also, both samples from patient 58 had a unique *msp*3α genotype (H) with a fragment size of 1.5 kb. These rare genotypes did not cluster in time but rather were collected throughout the 10-year sampling period as were the more common genotypes. Given the rarity of these less common genotypes, it is likely that their presence in both the PI and RI infections of individual patients was due to relapse episodes.

**Table 2:** Sample size, performance measures and diversity parameters for the nine molecular markers used to type the recurrent sample pairs used in this study

**Marker**	***N***	**GFR (%)**	***N***_**a**_	**% polyclonal**	***H***_**E**_
	(PI/RI)	(PI/RI)	(PI/RI)	(PI/RI)	(PI/RI)
*msp3α*	49/56	27.1/25.3	8/8	5.7/5.3	0.743/0.739
*csp* repeat	69/74	1.4/1.3	2/2	5.7/5.3	0.492/0.505
MS 40	62/69	0/0	5/5	0/0	0.616/0.59
MS 92	62/69	0/0	8/9	1.9/1.9	0.754/0.806
MS 116	61/68	1.6/1.4	12/11	0/0	0.822/0.744
MS 33	58/69	6.5/0	7/8	3.7/1.9	0.706/0.63
MS 176	49/63	21.0/8.8	6/6	0/0	0.692/0.747
MS 315	59/67	4.8/2.9	5/5	0/3.8	0.593/0.62
MS 206	50/57	19.4/17.4	10/10	0/0	0.825/0.831

*PI,* primary infection*; RI,* recurrent infection.

*N*, number of samples; GFR, genotype failure rate, *N*_a_, number of alleles,

*H*_E_,effective heterozygosity.

### Microsatellite variation

**Figure 3:**
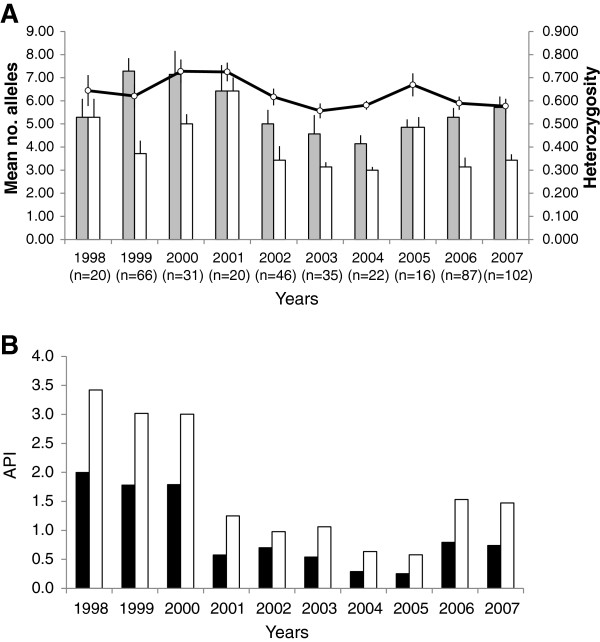
**Microsatellite diversity and *Plasmodium vivax *infection rate in southern Mexico during a 10-year period.A**. Pattern of allelic variation for 7 microsatellite loci using 474 bloodspot samples collected over 10 years. Grey, mean number of alleles (Na); white, mean number of alleles ≥ 5%; black line, mean effective heterozygosity (*H*_e_ = [n/(n-1)]×1- ∑*p*_i_^2^), shows a slight but persistent decrease over the time frame (R^2^ = 0.3109). **B**. Number of malaria cases per 1,000 people during a 10 year period across the entire study area (black bars), and within villages with positive malaria cases only (white bars). API: number of cases per year per 1,000 people.

Among the recurrent sample pairs, a subset of 62 primary (PI) and 69 recurrent (RI) infections were successfully genotyped using MS markers (Table 2 and Additional file 1). In addition, 326 non-recurrent samples were genotyped at 4–7 MS markers to obtain baseline information on population diversity (Table 3). Of these, 312 were successfully typed for all microsatellite markers. The number of alleles ranged from 6−15 for the baseline samples and from 5−12 for the recurrent samples (Tables 3 and 2). Effective heterozygosity (*H*_e_) for the seven MS used in this study ranged from 0.555-0.818 (Table 3). MS *H*_e_ declined slightly over the 10 year period (Figure 3A). Given the decline in the number of malaria cases in the region starting in 2001, it is perhaps surprising that diversity did not show a steeper decline in later years, although this may be explained by an increase in sample size (Figure 3). When the seven MS loci are used in combination as we have done here, the likelihood that two infections have the same seven-locus genotype by chance is negligible (πP_i_ = 1.39 × 10^-4^). Indeed, more than one MS haplotype could be found in parasite isolates with identical *csp*-*msp3α* genotypes. For most of the patients with rare *msp3α* genotypes D, E and G (patient 7, 23, 38, 43, 46 and 66), where relapse rather than re-infection was suspected, heterologous microsatellite alleles were found in the RI samples. Two of six patient samples had one new microsatellite allele in the RI (patients 23 and 43), they also had low frequent MS alleles (Additional file 1). If we define these pairs of infections as homologous, based on the rare but identical *msp3α* alleles and that 1–2 single unit differences in microsatellite alleles between infections could be due to DNA replication slippage within the patient.

**Table 3:** Sample size, performance measures and diversity parameters for the seven microsatellite markers used to type 326 baseline, non-recurrent samples collected between 1998 and 2007

**Marker**	***N***	**GFR (%)**	***N***_**a**_	**% polyclonal**	***H***_**E**_
MS 40	321	1.5	6	2.15	0.555
MS 92	325	0.3	11	1.2	0.743
MS 116	326	0	15	0.8	0.762
MS 33	326	0	12	1.2	0.686
MS 176	319	2.2	8	0.0	0.665
MS 315	324	0.6	8	1.2	0.63
MS 206	323	0.9	15	-	0.818

*N*, number of samples; GFR, genotype failure rate, *N*_a_, number of alleles,

*H*_E_,effective heterozygosity.

### Relapse criteria based on microsatellite and *csp-msp3* genotypes

**Figure 4:**
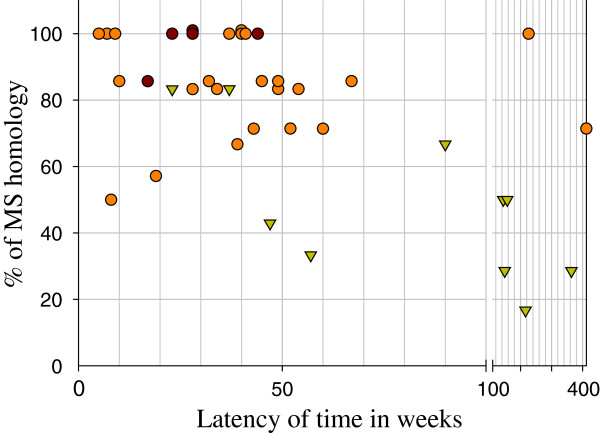
**Concordance between *P. vivax cspr-msp3α *and microsatellite patterns for primary and recurrent infections with different latency periods (n=39 pair samples).** Each data point indicates one sample pair (PI and RI). Circles indicate paired infections with homologous *cspr-msp3α* genotypes. Brown circles, rare *msp3α* genotypes (D, E, G); orange circles, common *msp3α* genotypes (A, B, C). Green triangles indicate paired infections with heterologous *cspr-msp3α* genotypes.

Thirty nine paired samples produced genetic marker information for all three genetic marker sets. Among the 39 pairs, ten had heterologous *cspr* and/or *msp3α* genotypes and all seven of them had three or more heterologous MS (patients 28, 29, 30, 37, 63, 65, 67), another one had two of six heterologous MS (patient 53) and only two pairs had 5 of 6 homologous microsatellites (patients 3 and 18) (Figure 4, Table 4 and Additional file 1). In contrast, 29 patients had the same *cspr-msp3α* genotype and > 80% homology of six or seven MS. In particular, 14 pairs of samples (patients 6, 7, 9, 17, 20, 21, 34, 36, 38, 45, 46, 61, 63RI2, 66) had the same alleles across all three-marker sets. However, four sample pairs with homogeneous *cspr-msp3α* genotypes had two of six (patient 25), or two of seven (patients 8, 19RI1, 42) heterologous MS. A single patient with homogeneous *cspr-msp3α* genotypes had three of six heterologous MS (patient 59). In spite of this, high agreement across marker sets suggests a low margin of error in accepting homology by either the same *cspr-msp3*genotypes and/or microsatellites (Chi-square*; p=0.003*).

**Table 4:** **Molecular classification of recurrent *P. vivax *carrying single genotype infections**

**Interval time in weeks**	**Genetic homology:**				**Genetic heterology:**	**N total**
	**By all three markers (N)**	*By*cspr*	*By*MS*	**Subtotal**	**By any of the three set of markers (N)**	
		***+msp3α***	**(N)**			
		**(N)**			**Subtotal**	
5-13	**4**	2	1	7	1**	8
14-26	**2**	1	-	3	**3**	6
27-40	**9**	-	7	16	**3**	19
41-52	**5**	1	3	9	**3**	12
53-69	**2**	-	-	2	**3**	5
70-104	**1**	-	-	1	**2**	3
≥105	**1**	-	-	1	**12**	13
N total	**24**	4	11	**39**	**27**	**66**

*only these marker results were obtained; **msp3α genotype J; N, number of recurrent infections. All markers correspond to *csp* (circumsporozoite gene), *msp3α* (merozoite surface protein 3α gene) and MS (microsatellite). Time-interval of homologous paired samples of rare *msp3α* genotypes (D, E, G, H, J) were within 14 and 52 weeks.

### More homologous genotypes from patients with short interval between PI and RI

The average duration between PI and RI for those homologous RIs (by *cspr*/*msp3α/MS*; n=22) was 41.5 ± 44.3 weeks, which was significantly different from those classified as heterologous RIs by *cspr* and/or *msp3α* (128.4 ± 104 weeks; n=10) (*t*-test; *p=*0.0022). Indeed, sample pairs with the same *cspr-msp3α* genotypes and a high degree of homology among microsatellite markers also had shorter recurrent intervals than those with heterologous *msp3α* or microsatellites (Figure 4, Table 4 and Additional file 1). The majority (76.6%) of paired samples with a latency period between five and 52 weeks were considered genetically homologous. The frequency of homologous recurrences declined gradually as the time interval between infections increased to 53–104 weeks and greater (total of 17.4%), (Table 4). The ratio of the number of homologous versus heterologous recurrences was higher within 52 weeks (4.5) than > 52 weeks (0.35) (Table 5). Homologous recurrent episodes occurred in people of both sexes and all ages, and originated from different locations throughout the transmission area (Additional files 1 and 2).

**Table 5:** Numbers and ratios of homologous and heterologous infections during low and high transmission seasons

**Group**	**# Hom**	**#Het**	**Ratio**	*P-value
PI_LTS	16	8	2.00	0.957
PI_HTS	35	18	1.94	
RI<52W	41	9	4.50	0.0001
RI>52W	6	17	0.35	

Hom, homologous infections; Het, heterologous infections;

Ratio, ratio of homologous infection over heterologous infection;

PI_LTS, Primary infection during the low transmission season; PI_HTS, primary.

infection during the high transmission season; RI < 52W, recurrent infection within 52 weeks of the primary infection; RI > 52W, recurrent infection at or after 52 weeks.

following the primary infection.

* Chi-square; IC 95%.

We observed more than two episodes of *P. vivax* malaria during the study period in five patients, including two patients who suffered three symptomatic malaria episodes (patients 19 and 43; Additional file 1). In both cases, parasites collected from all three episodes appear to be homologous based on our criteria for the marker set, although for patient 43 some data was missing (this episode was not included in the previous analysis) (Additional file 1). These putative homologous infections had latency periods of 27 and 43 weeks (patient 43), and 43 and 60 weeks (patient19). Interestingly, patient 51, also with three infections, showed a mixed PI and two different RI at 34 and 46 week intervals. Although MS data was not obtained for the PI, based on our criteria for *cspr*-*msp3α*, the first but not the second R1 was likely homologous to the PI. Patient 5 suffered 4 malaria episodes, two in 2002 with the same *cspr-msp3α* genotype (13 weeks apart), and the other two episodes in 2004 and 2005 (15 weeks apart); the third and fourth episodes were identical to each other but differed from the first and second episodes in their *msp3α* alleles (B versus A). There were also six polyclonal infections detected by *cspr-msp3α* in the PI only (patients 10, 26, 32, 35, 51, 70) that corresponded to 75% of the total detected, while the other two (25%) were in the RI only (patients 2, 52) (Additional file 1).

According to the transmission intensity estimated for the study region, 71.4% (50 of 70 patients) of primary infections occurred during the high transmission season. In areas of seasonal transmission, relapses can occur immediately prior to the initiation of malaria transmission each year or during active transmission [35-38]. We observed both incidences in our study. There were no differences in the ratios between the number of homologous and heterologous recurrences during low versus high transmission seasons (*p=0.957*) (Table 5) and were similarly distributed in the region (Additional file 3). In addition, no obvious correlation was observed between any genotypes and latency period; the *msp3α* A, B and C genotypes were found in relapses of short (5–26 weeks), long (27–52 weeks) or even late (over 52 weeks) latency periods. Similarly, patients infected with low frequency genotypes *cspr* vk210 and *msp3α*-D, E, G, H and J showed recurrence within 17–44 weeks, although the relatively shorter latency periods were likely associated with homologous relapses, compared with heterologous recurrences (relapse or re-infections). Heterologous and mixed genotype recurrent infections occurring during the lowest transmission season are likely relapses (e.g. patients 1, 2, 3 and 5).

## Discussion

Relapse episodes can be caused by parasites that are either homologous or heterologous to the primary infections. If a patient is infected with a single parasite genotype, the relapse will have the same genotype as the PI [17]. If, however, a patient is infected with more than one parasite genotype, the relapse could result from any one of those genotypes. Many factors may contribute to the latency and frequency of relapse, including the number of sporozoites inoculated, host immunity, co-infection with other parasites such as *Plasmodium falciparum*, drug treatment, and the genetic composition of *P. vivax* strains [5].

A better understanding of the mechanism of relapse and the development of methods to distinguish relapse from new infections or recrudescence will greatly facilitate efforts to control and eliminate *P. vivax* malaria. PQ is currently the only licensed drug that has been shown to be effective in treating hypnozoites to prevent relapse, making treating *P. vivax* malaria and interrupting transmission more difficult. In southern Mexico, the current antimalarial guideline is designed to suppress recurrent infections only if the drugs are administered to patients at the onset of the blood infection. However, putative relapse infections were detected within and between the three-month timeframe of the single dose administration strategy, warranting reconsideration of the guidelines.

Several studies have employed genetic markers to facilitate identification of *P. vivax* relapse infections [17,26,30,39,40], and WHO has recommended the use of at least one polymorphic marker to aid distinction between homologous and heterologous infections [10]. In this study, we used two types of polymorphic markers, PCR-RFLP size polymorphism in *cspr* and *msp3a* and a set of seven microsatellites, to develop criteria for identifying putative relapse infections in southern Mexico. While only two *cspr* genotypes (vk210 or vk247) were found in our samples, eight alleles were detected for *msp3a*. Genotyping results from the combination of these two genetic markers appeared to be consistent with those obtained using microsatellites, e.g. sample pairs with the same *cspr-msp3a* genotype also had highly similar microsatellite alleles. Recurrent infections in this low transmission, pre-elimination setting [41,42] were considered being relapses if: *1)* the PI and RI had exactly the same *cspr*-*msp3a* genotypes and > 80% homologous microsatellite alleles; *2)* the PI and RI had the same rare *cspr-msp3a* genotype, and *3)* the heterologous RI sample occurred during a non-transmission period when the chance of a new infection was vanishingly small. These criteria allow us to define relapses with a certain degree of confidence, although there is still a possibility of the appearance of new infections with the same genotype during local outbreaks (rapid expansion of parasites with the same genotype), as well as the possibility that mutations can occur during parasite replication within a host, particularly for MS markers that have relatively high mutation rates due to replication slippage. In a recent study, an infection with identical *cspr-msp1-msp3 and MS* genotypes (1–2 MS loci that differs by a single repeat unit) or identical *cspr-msp1-msp3* were considered as homologous infection [43]. Our first criterion was similar to those employed by Kim *et al.*[43]. Because our samples were from a low transmission region, we also considered annual transmission rate and the relatively low possibility of infecting parasites with multiple genotypes in determining relapse or re-infection. However, even though we used nine molecular markers to classify infections in this study, our classifications might miss some heterologous relapses using these criteria. Finally, the methods employed here, compared with, for example, deep-sequencing of all infections, may not identify all genotypes in either the PI or RI.

In southern Chiapas, Mexico, transmission mainly occurs on temperate zone hillsides and is seasonal, once per year generally lasting for 4–6 months; it begins after the rain ends and continues throughout the dry season until the onset of the next rainy season (January-June). Southern Mexico is also a region with low transmission intensity and thus provides an ideal setting for studying relapse in the field because of the low mixed genotype infection rate (< 10%) and the low likelihood that a patient will receive more than one infective bite within one year [19,35]. Our study of 76 recurrent symptomatic infections caused by *P. vivax* in this region showed a large percentage of relapse episodes occurring within the first 52 weeks, based on our definition of relapse, with a majority of these occurring between 27–52 weeks. Most parasites causing recurrent infections > 52 week after the primary episode were genetically different from the parasites in the PI, suggesting heterologous relapse or re-infections. A few recurrent infections with late intervals (60, 67, 81, and 226 weeks) were also considered to be relapses based on either genetic homology between the PI and RI, and/or occurrence of the RI during the time of year when no known transmission was occurring. Similarly, in El Salvador, 70% of observed recurrent infections happened within 12 months of the PI, and in the absence of mosquito transmission, and were attributed to relapse [35]. However, it is possible that some heterologous recurrences detected in the high transmission season may be relapses, and some homologous recurrences may correspond to re-infections because the resolution of using a limited number of genetic markers may not be sufficient to identify mixed genotypes or because recurrence infections were derived from parasites of same genotypes (outbreaks).

We found no association between *P. vivax* genotypes and relapse latency, but we cannot entirely rule out this possibility because our sample size was relatively small. Relapse, as defined in the literature, conforms to two main patterns: short-latency with relapses occurring mainly within the first 3–6 months after primary infection, as represented by the tropical strain "Chesson" isolated from the Pacific Coast of Asia and those *P. vivax* parasites found in Thai and Vietnamese [44]. In regions with stable malaria transmission and mild climate, parasites typically exhibit short-latency relapse, as seen in Thailand [45], Colombia [46], French Guyana [47] and Brazil [48,49]. In contrast, relapse with a relatively long latency period (7 to 14 months) is represented by the template strain "St Elizabeth" isolated from the Atlantic coast [5,50,51]. These criteria are similar to those proposed by Adak based on several *P. vivax* relapse studies in India [32]. The relatively long-term relapses detected here are consistent with relapses observed in other temperate zones [5,6,52]. During 2004–2005, malaria transmission in southern Mexico declined precipitately, with only a few malaria cases reported. This reduction did not lead to a concomitant reduction in parasite genetic diversity based on our microsatellite data (Figure 3) but may have selected for parasites capable of relapsing for longer intervals. In contrast, Hurricane STAN in October, 2005, created an environment that greatly increased parasite transmission, and may have altered relapse patterns in subsequent years. Increases in rainfall can have a dramatic impact on parasite transmission and, in theory, relapse patterns. For example, the incidence of mixed-genotype infections would be expected to increase due to increased parasite transmission. In addition, under conditions of increased transmission, hypnozoite-infected patients are more likely to receive additional bites from infected mosquitoes [36]. In Central America, relapses were reported to occur between 3 and 10 months, with the relapse interval varying between parasite strains and subjects, perhaps indicating adaptation to the unique local ecological environment, including transmission intensity and length of the dry season [8,19]. Late interval relapses appear to be less common, but have been documented in other regions, with intervals ranging from between one and two years [32,53], up to four years [54] and even twenty years [55]. In our study, we documented one putative relapse with a 226-week interval (> 4 years) containing a single haplotype in both PI and RI samples, and exhibiting perfect genetic homology across all genetic markers. Heterologous relapses were rarely observed in this study, and this may reflect a low rate of mixed genotype infections, typical of a low transmission area. With the exception of patient 2, all relapse episodes were produced by a single *P. vivax* genotype supporting the clonal activation of hypnozoites [18].

## Conclusions

In summary, our study represents the first investigation of *P. vivax* relapse in southern Mexico using molecular markers, and contributes to the global understanding of relapsing malaria. We demonstrate that a large number of putative relapse infections occurred within 52 weeks (one year) of the initial infection, and were largely homologous to the primary infection. They were located mainly in the foothills, which is consistent with observations from other regions with similar temperate zone climate conditions. Genetic markers when analyzed in combination with transmission information can provide a useful tool to characterize recurrent infections.

## Abbreviations

CQ: Chloroquine; PQ: Primaquine; PI: Primary infection; RI: Recurrent infection; csp: Circumsporozoite protein; msp3α: Merozoite surface protein 3 alpha; MS: Microsatellites; PCR: Polymerase chain reaction; RFLP: Restriction fragment length polymorphism

## Competing interests

The authors declare no competing interests.

## Authors’ contributions

LGC participated in the conception and design of the study, analysis and interpretation of data, preparing tables and figures, and manuscript writing; JM participated in microsatellite analysis and manuscript writing; GC and FSV carried out the PCR-RFLP analysis of genes *csp* and *msp3α*; MS participated in patient follow-up in the field and collection of *P. vivax* samples, and preparation of figures; DJ participated in microsatellite analyses; preparing figures and tables, interpretation of data and manuscript writing; XS participated in interpretation of data and manuscript writing; RT and VCH prepared a data base of the 10-year malaria case report and data analysis. All authors read and approved the final manuscript.

## Supplementary Material

Additional file 1 **Data of *P. vivax *primary and recurrent infections and genetic results.** Click here for file

Additional file 2 **Geographic distribution of *P. vivax recurrent *episodes in Southern Chiapas, Mexico.** Each circle correspond to one village. Patients with single and genetic homologous primary and recurrent infections are indicated in dark blue. Those with mixed primary infection with a genetic related or unrelated single recurrent infection are in blue and bright blue, respectively. Heterologous recurrent episodes are indicated in white. * indicate one mixed recurrent infection, likely a relapse. Click here for file

Additional file 3 **Temporal distribution of *P. vivax *homologous and heterologous recurrent single infections.** Brown arrows indicate homologous PI and RI genotypes and blue arrows indicate heterologous PI and RI genotypes. Round spot indicate primary blood infection and the arrow head indicate the recurrent blood infection. Thick arrows indicate rare *msp3α* genotypes. Patient number is indicated at the arrow side. Red line indicate cut-off value for low transmission setting. (PPTX 77 kb) Click here for file
