# Supplementary material for: Molecular and epidemiological characterization of Plasmodium vivax recurrent infections in southern Mexico
Source: Parasit Vectors. 2013 Apr 18;6:109. doi: 10.1186/1756-3305-6-109 (PMC3637411; doi:10.1186/1756-3305-6-109)
Supplement: Additional file 1 — Data of P. vivax primary and recurrent infections and genetic results. [file 1756-3305-6-109-S1.docx]

Additional file 1. Data of *P. vivax* primary and recurrent infections and genetic results.

| Patient  No. | |  | Latency of time in weeks | Date of sample collection Month, year | *cspr*  type | PCR-RFLP  *msp3α genotype* | Microsatellites:  nucleotide length | | | | | | | | | | | | Likely score | |
| --- | --- | --- | --- | --- | --- | --- | --- | --- | --- | --- | --- | --- | --- | --- | --- | --- | --- | --- | --- | --- |
|  |  | Age/  sex |  |  |  |  |  |  |  |  |  |  |  |  |  |  |  |  |  |  |
|  |  |  |  |  |  |  | 40 | | 92 | 116 | 33 | | 176 | | 315 | | 206 | |  |  |
|  |  |  |  |  |  |  | 4m | | 3m | 3m | 3m | | 2m | | 2m | | 3m | |  |  |
| 1 | PI | 30/F |  | Feb, 2004 | Vk247 | A | ND | | | | | | | | | | | |  | |
|  | RI |  | 18 | June, 2004 | Vk247 | **B** |  |  |  |  |  |  |  |  |  |  |  |  | Heterologous | |
| 2 | PI | 79/M |  | Feb, 2003 | Vk210 | C | ND | | | | | | | | | | | |  | |
|  | RI |  | 40 | Nov, 2003 | Vk210+ Vk247 | C |  |  |  |  |  |  |  |  |  |  |  |  | Likely Homologous^1^ | |
| 3 | PI | 39/F |  | Jan, 2004 | Vk247 | A | 156 | 123 | | 197 | | 143 | | 118 | | 212 | | - | |  |
|  | RI |  | 23 | July,2004 | Vk247 | **B** | 156 | 123 | | **179** | | 143 | | 118 | | 210 | | 181 | | Heterologous |
| 4 | PI | 46/F |  | Dec, 2004 | Vk210 | C | 160 | 105 | | 185 | | 149 | | - | | - | | - | |  |
|  | RI |  | 10 | Mach, 2005 | Vk210 | C | 160 | 105 | | 185 | | 149 | | 138 | | 200 | | 217 | | Likely Homologous^1^ |
| 5 | PI | 21/M |  | Sept, 2002 | Vk247 | A | ND | | | | | | | | | | | |  | |
|  | RI1 |  | 13 | Dec, 2002 | Vk247 | A |  |  |  |  |  |  |  |  |  |  |  |  | Likely Homologous^1,2^ | |
|  | RI2 |  | 117 | Nov, 2004 | Vk247 | B |  |  |  |  |  |  |  |  |  |  |  |  | Likely heterologous relapse^1^ | |
|  | RI3 |  | 132 (15)^4^ | March,2005 | Vk247 | B |  |  |  |  |  |  |  |  |  |  |  |  |  | |
| 6 | PI | 9/F |  | May, 2006 | Vk247 | A | 156 | 114 | | 179 | 143 | | - | | 200 | | - | |  | |
|  | RI |  | 81 | Feb, 2007 | Vk247 | A | 156 | 114 | | 179 | 143 | | 140 | | 200 | | 220 | | Likely Homologous | |
| 7 | PI | 41/F |  | Feb, 2002 | Vk210 | E | 164 | 102 | | 191 | 152 | | - | | 200 | | 214 | |  | |
|  | RI |  | 28 | Sept, 2002^1^ | Vk210 | E | 164 | 102 | | 191 | 152 | | 132 | | 200 | | 217 | | Homologous | |
| 8 | PI | 63/M |  | Jan, 2007 | Vk210 | B | 156 | 102 | | 197 | 140 | | 118 | | 212 | | 202 | |  | |
|  | RI |  | 19 | May, 2007 | Vk210 | B | 156 | 102 | | **179** | 143 | | 118 | | 212 | | **211** | | Heterologous^3^ | |
| 9 | PI | 16/M |  | May, 2002 | Vk210 | B | 156 | 114 | | 179 | - | | 118 | | 210 | | 181 | |  | |
|  | RI |  | 7 | July, 2002 | Vk210 | B | 156 | 114 | | 179 | 143 | | 118 | | 210 | | 181 | | Homologous | |
| 10 | PI | 43/F |  | March, 2007 | Vk247 | A+C | ND | | | | | | | | | | | |  | |
|  | RI |  | 8 | May, 2007 | Vk247 | C |  |  |  |  |  |  |  |  |  |  |  |  | Likely Homologous | |
| 11 | PI | 21/M |  | June, 2003 | Vk247 | B | ND | | | | | | | | | | | |  | |
|  | RI |  | 44 | May, 2004 | Vk247 | B |  |  |  |  |  |  |  |  |  |  |  |  | Homologous^1,2^ | |
| 12 | PI | 47/F |  | April, 2002 | Vk247 | A | ND | | | | | | | | | | | |  | |
|  | RI |  | 95 | Feb, 2004^1^ | Vk247 | **B** |  |  |  |  |  |  |  |  |  |  |  |  | Heterologous | |
| 13 | PI | 44/M |  | Nov, 2003 | Vk210 | C | 156 | 102 | | 185 | 140 | | 118 | | 212 | | 202 | |  | |
|  | RI |  | 113 | Jan, 2006^1^ | **Vk247** | **A** | 156 | **114** | | **197** | 143 | | **-** | | - | | 202 | | Heterologous | |
| 14 | PI | 53/F |  | Feb, 2003 | Vk210 | B | 148 | 102 | | 185 | 140 | | - | | - | | - | |  | |
|  | RI |  | 57 | March, 2004^1^ | **Vk247** | **C** | **156** | **123** | | **179** | 143 | | 118 | | 210 | | 181 | | Heterologous | |
| 15 | PI | 15/M |  | April, 2006 | Vk210 | C | 160 | 105 | | 182 | 149 | | - | | 200 | | 220 | |  | |
|  | RI |  | 49 | April, 2007 | Vk210 | C | 160 | 105 | | 182 | 149 | | 138 | | 200 | | **202** | | Homologous | |
| 16 | PI | 13/M |  | May, 2006 | Vk247 | A | 156 | 114 | | 179 | 140 | | 118 | | 200 | | 220 | |  | |
|  | RI |  | 34 | Jan, 2006^1^ | Vk247 | A | 156 | 114 | | 179 | 140 | | **138** | | 200 | | - | | Homologous | |
| 17 | PI | 24/F |  | March, 2005 | Vk210 | C | 160 | 105 | | 185 | 143 | | - | | 200 | | - | |  | |
|  | RI |  | 28 | Nov, 2005 | Vk210 | C | 160 | 105 | | 185 | 143 | | 130 | | 200 | | 220 | | Homologous | |
| 18 | PI | 18/M |  | April, 2004 | Vk247 | A | 156 | 105 | | 197 | 143 | | - | | 200 | | 220 | |  | |
|  | RI |  | 37 | Jan, 2005^1^ | Vk247 | **C** | 156 | 105 | | **185** | 143 | | 130 | | 200 | | 220 | | Heterologous | |
| 19 | PI | 19/M |  | Jun, 2006 | Vk210 | C | 160 | 105 | | 185 | 149 | | 130 | | 200 | | 220 | |  | |
|  | RI1 |  | 43 | March, 2007 | Vk210 | C | 160 | 105 | | **179** | 149 | | **138** | | 200 | | 220 | | Heterologous^3^ | |
|  | RI2 |  | 60 | July, 2007^1^ | Vk210 | C | 160 | 105 | | 185 | 143 | | **138** | | 200 | | 220 | | Homologous | |
| 20 | PI | 28/M | 226 | Feb, 1999 | Vk247 | A | 156 | 114 | | 200 | 143 | | 138 | | 200 | | 217 | |  | |
|  | RI |  |  | Jun, 2003 | Vk247 | A | 156 | 114 | | 197 | 143 | | 138 | | 200 | | 217 | | Homologous | |
| 21 | PI | 30/F | 40 | Feb, 2007 | Vk247 | A | 156 | 114 | | 179 | 143 | | 138 | | 200 | | 220 | |  | |
|  | RI |  |  | Nov, 2007^1^ | Vk247 | A | 156 | 114 | | 179 | 143 | | - | | 200 | | 220 | | Homologous | |
| 22 | PI | 62/F | 67 | May, 2006 | Vk210 | C | 156 | 153 | | 182 | 140 | | 134 | | 200 | | 220 | |  | |
|  | RI |  |  | Nov, 2007^1^ | Vk210 | C | 156 | 153 | | 182 | 140 | | **140** | | 200 | | 220 | | Homologous | |
| 23 | PI | 12/M | 17 | May, 2007 | Vk210 | G | 164 | 114 | | 197 | 140 | | 132 | | 216 | | 211 | |  | |
|  | RI |  |  | August, 2007^1^ | Vk210 | G | 164 | 114 | | **179** | 140 | | 132 | | 216 | | 211 | | Homologous | |
| 24 | PI | 37/M | 10 | July, 2007 | Vk247 | A | 156 | 114 | | 185 | 143 | | 138 | | 200 | | 220 | |  | |
|  | RI |  |  | Oct, 2007 | Vk247 | A | 156 | 114 | | **179** | 143 | | 138 | | 200 | | 220 | | Homologous | |
| 25 | PI | 11/M | 39 | April, 2007 | Vk210 | B | 156 | 102 | | 197 | 140 | | 118 | | 212 | | 181 | |  | |
|  | RI |  |  | Jan, 2008 | Vk210 | B | 156 | 102 | | **179** | 140 | | - | | 212 | | **205** | | Heterologous^1,3^ | |
| 26 | PI | 11/F | 33 | April, 2007 | Vk247 | A+C | ND | | | | | | | | | | | |  | |
|  | RI |  |  | Dic,2007 | Vk210 | C |  |  |  |  |  |  |  |  |  |  |  |  | Likely Homologous^1^ | |
| 27 | PI | 45/M | 54 | May, 2006 | Vk210 | C | 160 | 105 | | 185 | 149 | | - | | 200 | | 220 | |  | |
|  | RI |  |  | Jun, 2007 | Vk210 | C | 160 | 105 | | 185 | 149 | | 138 | | **210** | | 220 | | Homologous | |
| 28 | PI | 6/F | 216 | April, 2002 | Vk247 | B | 156 | 123 | | 179 | 143 | | 118 | | 212 | | - | |  | |
|  | RI |  |  | June, 2006 | **Vk210** | **C** | 160 | **105** | | **182** | **149** | | **138** | | **200** | | 220 | | Heterologous | |
| 29 | PI | 53/M | 364 | May, 1999 | Vk247 | A | 156 | 114 | | 197 | 143 | | 138 | | 200 | | 220 | |  | |
|  | RI |  |  | June, 2006 | Vk247 | **B** | 156 | **129** | | **179** | 140 | | **118** | | **210** | | **181** | | Heterologous | |
| 30 | PI | 64/F | 47 | May, 2002 | Vk247 | B | 156 | 123 | | 179 | 143 | | 138 | | 212 | | 181 | |  | |
|  | RI |  |  | April, 2003 | **Vk210** | **C** | **148** | **102** | | **185** | 140 | | 134 | | 212 | | **205** | | Heterologous | |
| 31 | PI | 9/F | 35 | Nov, 1999 | Vk210 | G | 164 | 120 | | 197 | 131 | | - | | 212 | | 160 | |  | |
|  | RI |  |  | July, 2000 | Vk210 | -* | 164 | 120 | | 197 | 131 | | 118 | | 212 | | 160 | | Homologous | |
| 32 | PI | 57/M | 147 | March, 2005 | Vk210+ Vk247 | A+C |  |  | |  |  | |  | |  | |  | |  | |
|  | RI |  |  | Jan, 2008 | Vk210 | C | 156 | 102 | | 179 | 140 | | 118 | | 212 | | - | | Likely Homologous^1^ | |
| 33 | PI | 21/F | 52 | June, 2006 | Vk247 | B | 156 | 123 | | 179 | 143 | | 118 | | 212 | | 181 | |  | |
|  | RI |  |  | June, 2007 | Vk247 | B | 156 | 123 | | 179 | 143 | | 118 | | **200** | | **220** | | Heterologous^3^ | |
| 34 | PI | 36/F | 40 | April, 2007 | Vk210 | B | 156 | 102 | | 179 | 140 | | 118 | | 210 | | - | |  | |
|  | RI |  |  | Jan, 2008 | Vk210 | B | 156 | 102 | | 179 | 140 | | 118 | | 212 | | 205 | | Homologous | |
| 35 | PI | 10/M | 27 | Jan, 2007 | Vk210+ Vk247 | B |  |  | |  |  | |  | |  | |  | |  | |
|  | RI |  |  | July, 2007 | Vk247 | B | 156 | 114 | | 197 | 143 | | 118 | | 212 | | 205 | | Likely Homologous | |
| 36 | PI | 11/F | 5 | Jan, 2006 | Vk247 | B | 156 | 123 | | 179 | 143 | | 118 | | 210 | | 181 | |  | |
|  | RI |  |  | March, 2006 | Vk247 | B | 156 | 123 | | 179 | 143 | | 118 | | 212 | | 181 | | Homologous | |
| 37 | PI | 32/M | 144 | April, 1999 | Vk247 | A | 156 | 102 | | 197 | 143 | | 138 | | 200 | | 220 | |  | |
|  | RI |  |  | Jan,2002 | **Vk210** | **B** | 156 | 102 | | **179** | 140 | | - | | **212** | | **202** | | Heterologous | |
| 38 | PI | 16/M | 23 | Dic, 2007 | Vk210 | D | 164 | 126 | | 242 | 146 | | - | | 206 | | 211 | |  | |
|  | RI |  |  | May, 2008 | Vk210 | D | 164 | 126 | | 242 | 146 | | 132 | | 206 | | 211 | | Homologous | |
| 39 | PI | 34/F | 49 | Jun, 2007 | Vk210 | C | 156 | 153 | | 182 | 143 | | 134 | | 200 | | 220 | |  | |
|  | RI |  |  | May, 2008 | Vk210 | C | 156 | **120** | | 182 | 143 | | 134 | | 200 | | 220 | | Homologous | |
| 40 | PI | 21/F | 249 | April, 2003 | Vk247 | C |  |  | |  | ND | |  | |  | |  | |  | |
|  | RI |  |  | Feb, 2008 | Vk247 | **B** |  |  | |  |  | |  | |  | |  | | Heterologous | |
| 41 | PI | 54/M | 28 | July, 2007 | Vk247 | B | 156 | 114 | | 179 | - | | 138 | | 200 | | 205 | |  | |
|  | RI |  |  | Feb, 2008 | Vk247 | B | 156 | 114 | | 179 | 143 | | 138 | | 200 | | **220** | | Homologous | |
| 42 | PI | 13/F | 412 | Feb, 2000 | Vk247 | A | 156 | 114 | | 197 | 143 | | 118 | | 200 | | 220 | |  | |
|  | RI |  |  | Jan, 2008 | Vk247 | A | 156 | 114 | | **179** | 143 | | **138** | | 200 | | 220 | | Heterologous^3^ | |
| 43 | PI |  |  | July, 1999 | Vk210 | - | 164 | 114 | | 233 | 140 | | 118 | | 200 | | 202 | |  | |
|  | RI1 | 39/M | 27 | Jan, 2000 | Vk210 | D | 164 | 114 | | **239** | 140 | | 118 | | 200 | | - | | Likely Homologous | |
|  | RI2 |  | 43 | May, 2000 | Vk210 | D | 164 | 114 | | **200** | 143 | | 118 | | 200 | | - | | Likely Homologous | |
| 44 | PI | 19/M | 6 | Jan, 1997 | Vk210 | C | 164 | 126 | | 218 | 146 | | 132 | | 200 | | 211 | |  | |
|  | RI |  |  | Feb, 1997 | Vk210 | - | 164 | 126 | | 218 | 146 | | 132 | | 200 | | 208 | | Homologous | |
| 45 | PI | 14/F | 41 | May, 1999 | Vk247 | A | 148 | 114 | | 197 | 143 | | 130 | | 200 | | 181 | |  | |
|  | RI |  |  | Feb, 2000 | Vk247 | A | 148 | 114 | | 197 | 143 | | 130 | | 200 | | - | | Homologous | |
| 46 | PI | 38/F | 44 | Sept, 1999 | Vk210 | E | 164 | 102 | | 224 | 152 | | 132 | | 212 | | 172 | |  | |
|  | RI |  |  | Jun, 2000 | Vk210 | E | 164 | 102 | | 224 | 152 | | 132 | | 212 | | 172 | | Homologous | |
| 47 | PI | 28/M | 201 | May, 1997 | Vk247 | **-** | ND | | | | | | | | | | | |  | |
|  | RI |  |  | March, 2001 | **Vk210** | **-** |  |  |  |  |  |  |  |  |  |  |  |  | Heterogenous | |
| 48 | PI | 31/F | 216 | April, 1999 | Vk247 | -* | ND | | | | | | | | | | | |  | |
|  | RI |  |  | June, 2003 | **Vk210** | D |  |  |  |  |  |  |  |  |  |  |  |  | Heterologous | |
|  | PI | 53/M | 30 | Feb, 2001 | Vk247 | - | 148 | 114 | | 200 | 143 | | 130 | | 200 | | 202 | |  | |
| 49 | RI |  |  | Sept, 2001 | Vk247 | A | 148 | 114 | | 200 | 143 | | 130 | | 200 | | 202 | | Homologous | |
| 50 | PI | 34/M | 32 | May, 1997 | Vk210 | J | 156 | 102 | | 191 | 152 | | 118 | | 200 | | - | |  | |
|  | RI |  |  | Nov, 1997 | Vk210 | - | 156 | 102 | | 191 | 152 | | 118 | | 200 | | 172 | | Homologous | |
| 51 | PI | 30/F | 34 | Feb, 2005 | Vk210+ Vk247 | C |  |  | |  |  | |  | |  | |  | |  | |
|  | RI1 |  | 46 | Oct, 2005 | Vk247 | C | 156 | 114 | | 179 | 143 | | 118 | | 212 | | 202 | |  | |
|  | RI2 |  |  | Jan, 2006 | Vk210 | **B** | 160 | **105** | | **185** | **149** | | **138** | | **200** | | **220** | | Heterologous^1^ | |
| 52 | PI | 22/F | 164 | March,2003 | Vk247 | B |  |  | |  |  | |  | |  | |  | |  | |
|  | RI |  |  | May, 2006 | Vk247 | **A+C** |  |  | |  |  | |  | |  | |  | | Heterologous | |
| 53 | PI | 11/M | 90 | April, 2001 | Vk247 | A | 148 | 102 | | 197 | 143 | | 118 | | 212 | | - | |  | |
|  | RI |  |  | Jan, 2003 | **Vk210** | **C** | 148 | **126** | | **185** | 140 | | 118 | | 210 | | 202 | | Heterologous | |
| 54 | PI | 34/F | 29 | July, 2000 | Vk247 | -* | 156 | 114 | | 197 | 143 | | 138 | | 200 | | 220 | |  | |
|  | RI |  |  | Jan, 2001 | Vk247 | A | 156 | 114 | | 197 | 143 | | 138 | | 200 | | 217 | | Homologous | |
| 55 | PI | 46/M | 45 | Jul , 2000 | -* | -* | 156 | 105 | | 179 | 143 | | 118 | | 212 | | 204 | |  | |
|  | RI |  |  | May, 2001 | -* | -* | 156 | 105 | | 179 | 143 | | **138** | | 212 | | - | | Homologous | |
| 56 | PI | 29/M | 30 | March, 1999 | Vk247 | A | 148 | 114 | | 200 | 143 | | 130 | | 200 | | 181 | |  | |
|  | RI |  |  | Oct, 1999 | Vk247 | - | **156** | 114 | | 200 | 143 | | **138** | | 200 | | **217** | | Heterologous | |
| 57 | PI | 37/M | 30 | Dic, 2000 | Vk210 | **-** | 164 | 102 | | 194 | 134 | | 138 | | 212 | | 160 | |  | |
|  | RI |  |  | July, 2001 | Vk210 | **-** | **156** | 102 | | 194 | 134 | | 134 | | 210 | | 160 | | Homologous | |
| 58 | PI | 65/F | 26 | Aug, 2000 | Vk210 | H | ND | | | | | | | | | | | |  | |
|  | RI |  |  | Feb, 2001 | Vk210 | H |  |  |  |  |  |  |  |  |  |  |  |  | Homologous^2^ | |
| 59 | PI | 16/M | 8 | Jun, 2000 | Vk210 | J | 164 | 102 | | 188 | 152 | | 118 | | 200 | | 205 | |  | |
|  | RI |  |  | Aug, 2000 | Vk210 | J | 164 | **126** | | **251** | **137** | | 118 | | 200 | | **-** | | Heterologous | |
| 60 | PI | 1/F | 32 | Aug, 2001 | Vk247 | C | 156 | 102 | | 179 | 140 | | 118 | | 212 | | 202 | |  | |
|  | RI |  |  | March, 2002 | Vk247 | C | 156 | 102 | | 179 | 140 | | **130** | | 212 | | 202 | | Homologous | |
| 61 | PI | 25/F | 37 | May, 2002 | Vk210 | C | 148 | 102 | | 185 | - | | 118 | | 212 | | 205 | |  | |
|  | RI |  |  | Feb, 2003 | Vk210 | C | 148 | 102 | | 185 | 140 | | 118 | | 212 | | 205 | | Homologous | |
| 62 | PI | 17/F | 45 | May, 1999 | Vk247 | A | 152 | 114 | | 197 | 143 | | 138 | | 200 | | 220 | |  | |
|  | RI |  |  | March, 2000 | Vk247 | A | 152 | 114 | | 197 | 143 | | **130** | | 200 | | 220 | | Homologous | |
| 63 | PI | 20/F | 9 | May, 1999 | Vk247 | A | 156 | 114 | | 197 | 143 | | 130 | | 200 | | 220 | |  | |
|  | RI1 |  | 149 | July, 1999 | Vk247 | A | 156 | 114 | | 197 | 143 | | 130 | | 200 | | 220 | | Homologous | |
|  | RI2 |  |  | May, 2002 | Vk247 | B | 156 | **123** | | **179** | 143 | | **118** | | **212** | | **181** | | Heterologous | |
| 64 | PI | 46/M | 37 | April, 1999 | Vk247 | -* | 148 | 102 | | 197 | 143 | | 118 | | 212 | | 181 | |  | |
|  | RI |  |  | Jan, 2000 | Vk247 | A | 148 | 102 | | 197 | 143 | | 118 | | 212 | | 181 | | Homologous | |
| 65 | PI | 45/M | 157 | Feb, 1999 | Vk247 | A | 148 | 114 | | 179 | 143 | | 130 | | 200 | | 181 | |  | |
|  | RI |  |  | March, 2003 | **Vk210** | **C** | **160** | **123** | | **185** | 143 | | 130 | | 200 | | - | | Heterologous | |
| 66 | PI | 26/F | 28 | Oct, 1993 | Vk210 | E | 156 | 117 | | 215 | 140 | | - | | 198 | | 220 | |  | |
|  | RI |  |  | April, 1994 | Vk210 | E | 156 | 117 | | 215 | 140 | | 160 | | 198 | | 220 | | Homologous | |
| 67 | PI | 17/M | 57 | April, 1999 | Vk247 | **A** | 156 | 114 | | 200 | 143 | | 138 | | 200 | | 217 | |  | |
|  | RI |  |  | June, 2000 | Vk247 | **B** | 156 | **123** | | **179** | 143 | | - | | **212** | | **181** | | Heterologous | |
| 68 | PI | 39/M | 43 | Dec, 1999 | Vk210 | C | 148 | 135 | | 200 | 146 | | 140 | | 212 | | 202 | |  | |
|  | RI |  |  | Oct, 2000 | Vk210 | -* | 148 | 135 | | 200 | 146 | | 140 | | 212 | | 199 | | Homologous | |
| 69 | PI | 43/M | 197 | Aug, 1998 | Vk247 | -***** | 156 | 102 | | 182 | 140 | | 118 | | 210 | | 172 | |  | |
|  | RI |  |  | June, 2002 | Vk247 | - | 156 | 105 | | **179** | 143 | | **138** | | - | | - | | Heterologous | |
| 70 | PI | 50/M | 38 | July, 1999 | Vk210+ Vk247 | B |  |  | |  |  | |  | |  | |  | |  | |
|  | RI |  |  | March, 2000 | Vk247 | B | 156 | 102 | | 179 | 143 | | 130 | | 212 | | 205 | | Homologous? | |

m, indicate number of nucleotides per microsatellite unit (mer);

^1^indicates low transmission;

^2^given by *cspr and/or msp3α*;

^3^suggested by the detection of two of six or seven variable MS (above one repeat unit);

PI, primary infection; RI, recurrent infection; *cspr*, circumsporozoite repeat type. *msp3α, merozoite surface protein 3 alpha.* (-) indicates genotype failure and (-*) indicates insufficient sample
