# Supplementary material for: Molecular and epidemiological characterization of Plasmodium vivax recurrent infections in southern Mexico
Source: Parasit Vectors. 2013 Apr 18;6:109. doi: 10.1186/1756-3305-6-109 (PMC3637411; doi:10.1186/1756-3305-6-109)
Supplement: Additional file 2 — Geographic distribution of P. vivax recurrent episodes in Southern Chiapas, Mexico. Each circle correspond to one village. Patients with single and genetic homologous primary and recurrent infections are indicated in dark blue. Those with mixed primary infection with a genetic related or unrelated single recurrent infection are in blue and bright blue, respectively. Heterologous recurrent episodes are indicated in white. * indicate one mixed recurrent infection, likely a relapse. [file 1756-3305-6-109-S2.pptx]

## Slide 1
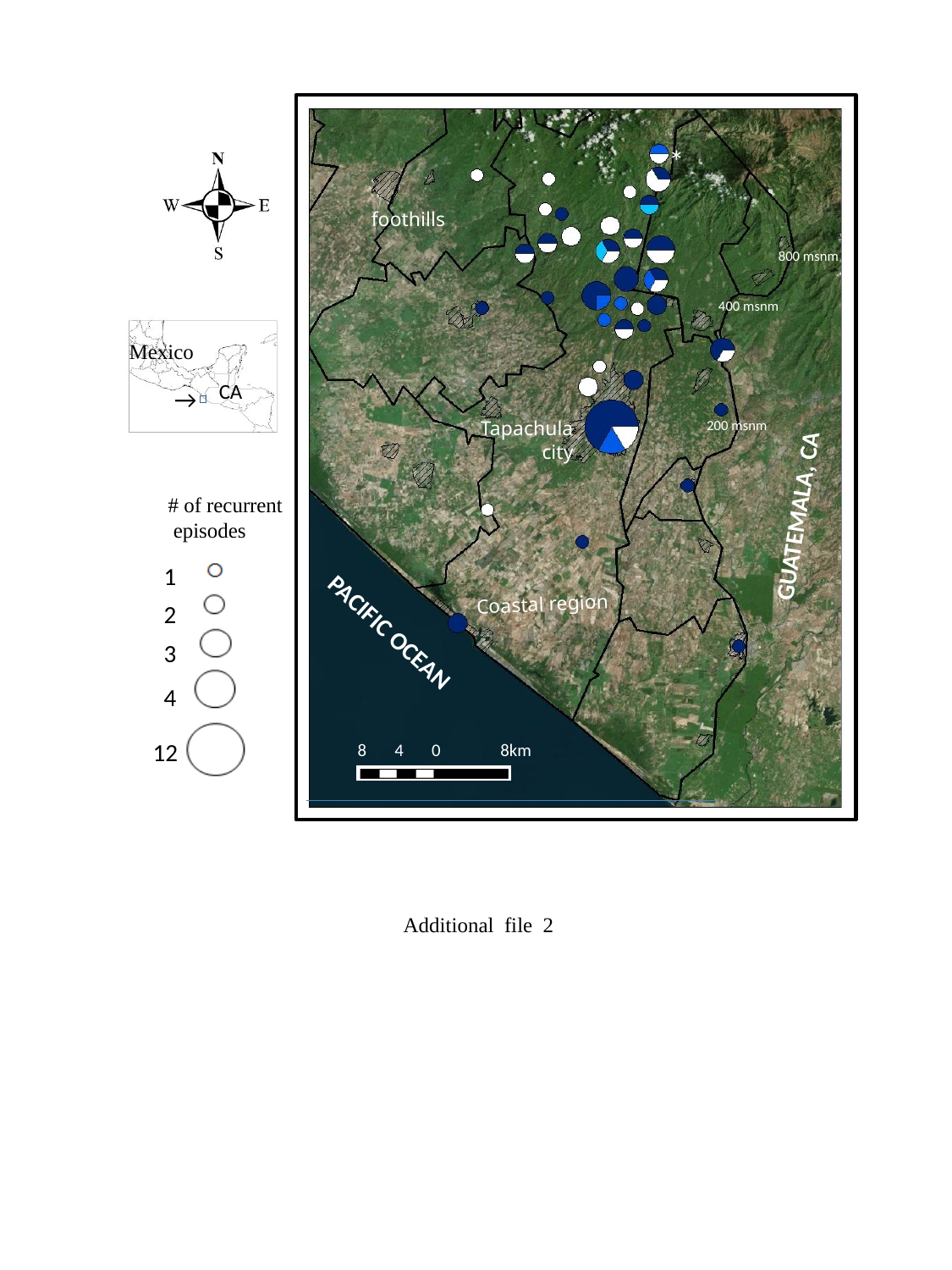

*
foothills
800 msnm
400 msnm
Mexico
CA
→
Tapachula
city
200 msnm
# of recurrent
 episodes
 1
 2
 3
 4
12
GUATEMALA, CA
Coastal region
PACIFIC OCEAN
8 4 0 8km
Additional file 2
