# Supplementary material for: Molecular and epidemiological characterization of Plasmodium vivax recurrent infections in southern Mexico
Source: Parasit Vectors. 2013 Apr 18;6:109. doi: 10.1186/1756-3305-6-109 (PMC3637411; doi:10.1186/1756-3305-6-109)
Supplement: Additional file 3 — Temporal distribution of P. vivax homologous and heterologous recurrent single infections. Brown arrows indicate homologous PI and RI genotypes and blue arrows indicate heterologous PI and RI genotypes. Round spot indicate primary blood infection and the arrow head indicate the recurrent blood infection. Thick arrows indicate rare msp3α genotypes. Patient number is indicated at the arrow side. Red line indicate cut-off value for low transmission setting. (PPTX 77 kb) [file 1756-3305-6-109-S3.pptx]

## Slide 1
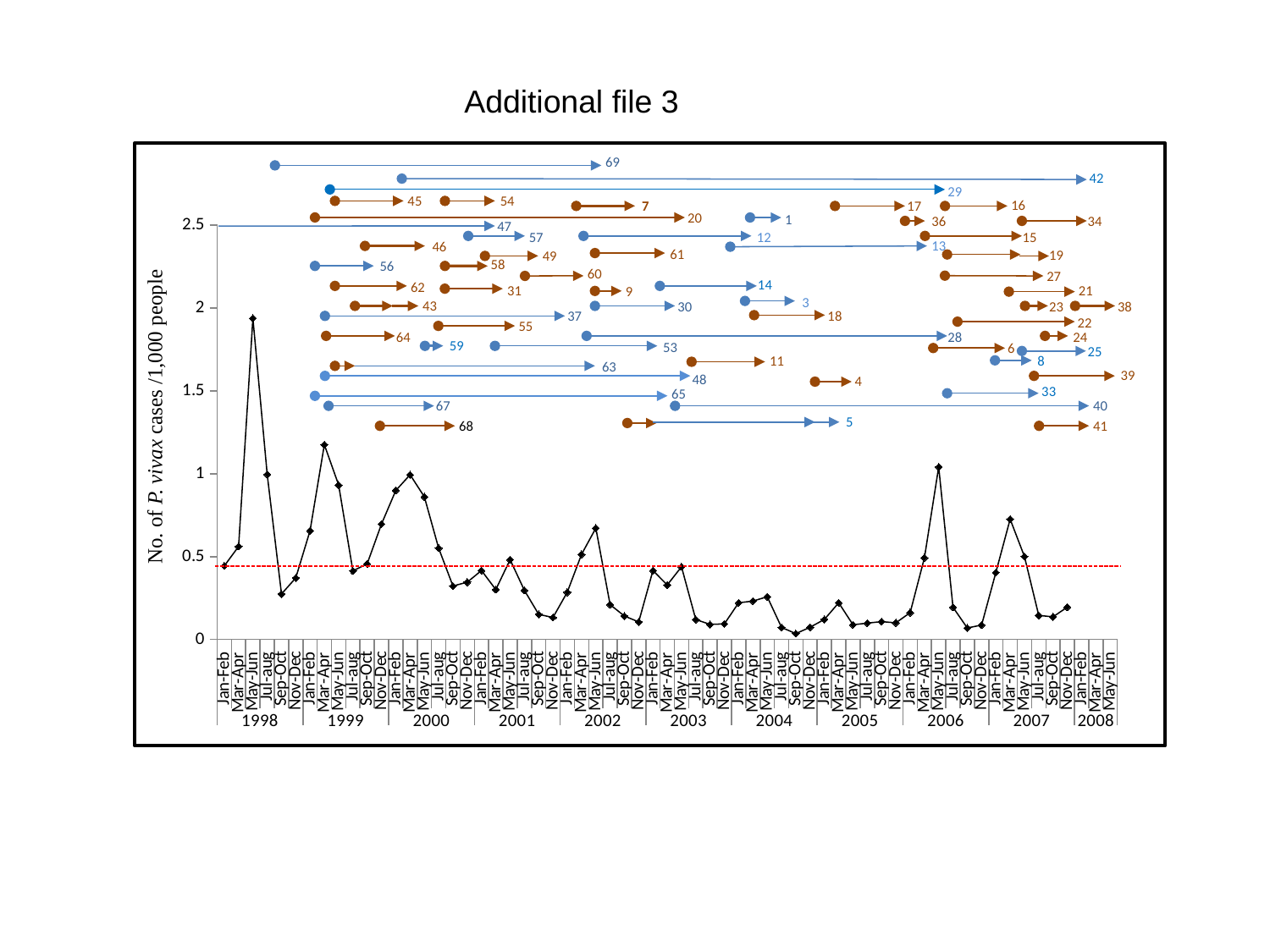

Additional file 3
69
42
29
45
54
16
17
7
20
1
### Chart
| Category | IPA (POB LOCALIDADES CON CASOS) |
|---|---|
| Jan-Feb | 0.443824499112351 |
| Mar-Apr | 0.5610549045296844 |
| May-Jun | 1.9381888478823028 |
| Jul-aug | 0.9959414717505073 |
| Sep-Oct | 0.27458546747260554 |
| Nov-Dec | 0.37136917219382 |
| Jan-Feb | 0.656007988203467 |
| Mar-Apr | 1.175405685500269 |
| May-Jun | 0.9317396242557147 |
| Jul-aug | 0.4136523118402767 |
| Sep-Oct | 0.45681916813229484 |
| Nov-Dec | 0.6965507512167596 |
| Jan-Feb | 0.8988569493355333 |
| Mar-Apr | 0.993771857291327 |
| May-Jun | 0.8607408157508336 |
| Jul-aug | 0.5510405320924717 |
| Sep-Oct | 0.3226908026855394 |
| Nov-Dec | 0.3455661681381767 |
| Jan-Feb | 0.416029003164792 |
| Mar-Apr | 0.3008639582072611 |
| May-Jun | 0.4815278749328952 |
| Jul-aug | 0.29649266616669867 |
| Sep-Oct | 0.1517623401752855 |
| Nov-Dec | 0.1326373438302242 |
| Jan-Feb | 0.2849601590077687 |
| Mar-Apr | 0.5136091404405625 |
| May-Jun | 0.6718190856217171 |
| Jul-aug | 0.21038174362801332 |
| Sep-Oct | 0.14138614981276434 |
| Nov-Dec | 0.10601374491249257 |
| Jan-Feb | 0.41596532977344397 |
| Mar-Apr | 0.3291011425045345 |
| May-Jun | 0.4384162214001918 |
| Jul-aug | 0.12051897966138322 |
| Sep-Oct | 0.09074821906620083 |
| Nov-Dec | 0.09432360542549378 |
| Jan-Feb | 0.22153755266645062 |
| Mar-Apr | 0.23162576015363104 |
| May-Jun | 0.2577984016499098 |
| Jul-aug | 0.07281406120426367 |
| Sep-Oct | 0.03550505947097461 |
| Nov-Dec | 0.07390505048638762 |
| Jan-Feb | 0.12110464739084362 |
| Mar-Apr | 0.2215368576517093 |
| May-Jun | 0.08866241087094487 |
| Jul-aug | 0.09870516766418708 |
| Sep-Oct | 0.10786856215701168 |
| Nov-Dec | 0.10066041988524713 |
| Jan-Feb | 0.16183563831845735 |
| Mar-Apr | 0.49299712473488994 |
| May-Jun | 1.0412508409566108 |
| Jul-aug | 0.19417401799345899 |
| Sep-Oct | 0.0695794156256813 |
| Nov-Dec | 0.08693742335779783 |
| Jan-Feb | 0.40410725706630857 |
| Mar-Apr | 0.7257797577292507 |
| May-Jun | 0.5007155195145726 |
| Jul-aug | 0.14516376546512544 |
| Sep-Oct | 0.1367820651356194 |
| Nov-Dec | 0.19526837915758924 |
| Jan-Feb | None |
| Mar-Apr | None |
| May-Jun | None |36
34
47
57
12
15
13
46
61
19
49
58
56
60
27
14
62
31
21
9
3
43
38
23
30
18
37
22
55
64
28
24
59
53
6
25
8
11
63
39
48
4
33
No. of P. vivax cases /1,000 people
65
67
40
5
68
41
